# Chronic rhinosinusitis: correlation of symptoms with computed tomography scan findings

**DOI:** 10.11604/pamj.2014.18.40.2839

**Published:** 2014-05-10

**Authors:** Enema Job Amodu, Ayotunde James Fasunla, Aliu Oyebamiji Akano, Abiodun Daud Olusesi

**Affiliations:** 1 Department of ENT, National Hospital, Plot 132, Central district, (Phase II), PMB 425, Garki-Abuja, Nigeria; 2 Department of Otorhinolaryngology, University College Hospital & College of Medicine, University of Ibadan, Ibadan, Oyo-State, Nigeria; 3 Department of Radiology, National Hospital, Plot 132, Central district, (Phase II), PMB 425, Garki-Abuja, Nigeria

**Keywords:** Computerized Tomographic Scan, chronic rhinosinusitis, nasal discharge, nasal obstruction, severity, symptoms

## Abstract

**Introduction:**

Symptomatology, nasal endoscopy and Computerised Tomographic (CT) scan have been used to diagnose chronic rhinosinusitis. The value of disease severity score in the assessment of chronic rhinosinusitis has not been well investigated. Hence, this study aims to correlate the pre-operative symptom severity score as well as overall disease severity scoreof patients with chronic rhinosinusitis with CT scan scores.

**Methods:**

This is a prospective study of 60 patients diagnosed clinically with chronic rhinosinusitis. Each patientsubjectively assessed his/her presenting symptoms and severity of disease on a visual analogue scale. The patients had CT scan of the paranasal sinuses which were graded and scored using Lund-Mackay grading system. The correlation study between severity of symptoms/disease severity and CT scores was performed. The level of statistical significance was considered at p < 0.05 and confidence interval at 95%.

**Results:**

All patients had more than one symptom with mean symptom severity scores highest for nasal discharge and nasal obstruction. There was a significant correlation between CT scores and nasal discharge (r = -0.132; p = 0.03)and nasal obstruction (r = 0.193; p = 0.049). No correlation with other symptoms. There wasno correlation between the overall disease severity scores and the Lund-Mackay CT scores (r = 0.195; p = 0.6).

**Conclusion:**

This study showed that CT scan scores can help clinicians to predict severity of symptom for nasal obstruction and discharge but not for other symptoms of chronic rhinosinusitis. However, there was no association of CT score with the overall disease severity score.

## Introduction

Chronic rhinosinusitis (CRS) is a common disease worldwide, constituting 78% of the rhinologic cases attending an otolaryngology clinic in West Africa [1]. Its burden on the quality of life of the affected individual is significant and the disease poses management challenges to the physicians. According to the diagnostic guidelineformulated by the Rhinosinusitis Task Force of the American Academy of Otolaryngology, Head and Neck Surgery (AAO-HNS) and revised by the Sinus and Allergy Health Partnership (SAHP), chronic rhinosinusitis can be diagnosed using clinical symptoms and signs which are categorized into minor and major criteria [2, 3]. The use of symptoms and signs alone to make diagnosis of chronic rhinosinusitis may not be adequate because of its overlap with other benign sinonasal diseases. The presenting symptoms may also not indicate the specific paranasal sinus that is affected and extent of disease spread. More recently, the position paper on rhinosinusitis and nasal polyps by the European Academy of Allergy and clinical immunology included the nasal endoscopyand/ or computerized tomography scan findings in the diagnosis [4]. The Lund-Mackay system of staging was recommended by the American academy of otolaryngology to stage chronic rhinosinusitis [5].

Computed Tomography (CT) scan of the paranasal sinuses is the gold standard diagnostic radiological tool for chronic rhinosinusitis. Because CT scan is not widely available in resource poor countries, it is often indicated after failedmedical treatment, when surgical treatment is plannedand if there is complication [6]. CT scanhas been shown to havethe advantage of concurrent evaluation of the nasal cavities,osteomeatal complex and paranasal sinuses. It is alsoreliable, accurate and effective atdemonstratingthe extent of disease spread and its associated complications [7]. Although publications on the correlation of symptoms of CRS to CT findings exist in the literature [8–12]but there is none on the diagnostic value of CT Scan in CRS in our environment where the weather is hot and highlyhumidwith probable increased predisposition to the disease [13, 14]. Studies have shown that clinical symptoms of CRS do not have good correlation with the CT scan stage [10, 11] while astudy has shown that preoperative CT scan stage may predict symptom improvement after endoscopic sinus surgical intervention [12]. This study therefore aimed to evaluate the value of CT scan in the management of chronic rhinosinusitis by correlating pre-operativesymptoms severity score as well as overall disease severity scoreof CRS with radiological findings on CT scan.

## Methods

This was a prospective hospital based study of all consecutive adult patients with clinical symptoms of CRS at the National Hospital, Abuja, Nigeria between January 2011 and December 2011. Ethical approval was obtained from institutional review board of the National Hospital, Abuja, Nigeria for the conduct of the study. Clinical diagnosis of chronic rhinosinusitis was made using the guideline by the Rhinosinusitis Task Force of the American Academy of Otolaryngology, Head and Neck Surgery (AAO-HNS) [2, 3]. The diagnosis was made if the patient presented with at least 2 major criteria or one major and 2 minor criteria for more than one hour on most days for more than 3 months. The major criteria included nasal obstruction or blockage, nasal discharge (Anterior or/& posterior), anosmia/ hyposmia and facial pain/ congestion /Pressure while minor criteria included headache, halitosis, ear ache, dental pain, cough and fatigue [5]. Patients with sinonasal mass, presence of co-morbidities such as diabetes mellitus, HIV infection and previous history of facial trauma or sinonasal surgeries were excluded from the study. A proforma was use to collect the patient's biodata and presenting clinical symptoms. The patients subjectively scored the severity of each presented clinical symptoms on a visual analogue scale calibrated from 0 to 10 where 0 means none, 1 means least severe and 10 means most severe. If the average total score is ≤ 4, the severity is scored mild/low; from 5 to 7 is moderate/intermediate while ≥ 8 is severe/high. The overall disease severity was also scored on a similar visual analogue scale by asking how troublesome was their diseases in affecting their daily routine activity and sleep. The scale was calibrated from 0 to 10 where 0 means not troublesome, 1 means least troublesome and 10 means most troublesome imaginable. A score of ≤4 ismild/low; from 5 to 7 is moderate/intermediate while ≥ 8 is severe/high.

All patients had CT scan of the paranasal sinuses doneon Aquilion 64- CT scanner (Toshiba™). Axial, coronal and sagittal reconstruction images of the paranasal sinuses and brain of the patients were obtained with 3.0mm section thickness, at 120 kV and 300 mA. Images were then transferred into a computer workstation with DICOM viewer for paranasal sinuses evaluation. All scans were evaluated using bone window (window width 2,000 Hounsfield units (HU); window level 500 HU) and soft tissue window (window width 200 HU; window level 40 HU). The radiological findings were graded according to the Lund-Mackay grading system [15]. The paranasal sinuses were maxillary, ethmoid, frontal and sphenoid sinuses. The ethmoid sinus was divided into anterior and posterior. For the paranasal sinuses, each side (right and left) was scored separately and the score was graded as follows; 0 if no abnormality; 1 if partial opacification, and 2 if total opacification is present. However, for the osteo-meatal complex, it was 0 if not occluded (patent) and 2 if occluded. The total score for both sides can range from 0 to 24. The anatomical variations observed were evaluated for their association with the disease severity.

Data obtained were collated and presented in tables, graphs and diagrams as appropriate. Statistical analysis was performed with Statistical Package for Social Sciences (SPSS) version 17 and correlation study between severity of symptoms/disease severity and CT findings was by Pearson correlation. Regression analysis was used to test the association among the variables. The CT score was used as dependent variable while the symptom severity score and disease severity score were the independent variables. The level of statistical significance was considered at p

## Results

Sixty patients diagnosed with chronic rhinosinusitis participated in the study. There were 36 (57%) males and 24(43%) females with a male to female ratio of 1.5:1. The patients’ ages range from 18years to 60 years (Mean = 35.6 ± 11.42). The Mean age for Male in the study was 36.6 years ± 11.9 while that of female was 34.2 ± 10.8. All the patients had more than one symptom with all of them presenting with nasal obstruction and nasal discharge. The mean symptom severity scores were higher for nasal discharge and nasal obstruction while the lowest mean symptom severity scores were forfatigue, ear pain/ pressure/ fullness and dental pain (Table 1).

**Table 1:** Average symptom severity score for each symptom

Symptoms	Number of patients with the symptom	Mean severity score
**Nasal obstruction/blockage**	60	6.2 (4 – 10)
**Nasal discharge**	60	6.1 (3 - 10)
**Facial**	9	0.5 (0 – 8)
**pain/pressure/fullness**	8	0.8 (0 – 7)
**Hyposmia/anosmia**	12	1.0 (0 – 9)
**Headache**	6	0.5 (0 – 8)
**Halithosis**	7	0.3 (0 - 6)
**Fatigue**	3	0.3 (0 – 7)
**Dental pain**	7	1.2 (0 – 7)
**Cough**	4	0.2 (0 – 6)

The overall disease severity score was mild/low in 16 (26.7%) patients; moderate/intermediate in 24 (40.0%) patients and severe/high in 20 (33.3%) patients.

The CT scans showed involvement of at least one paranasal sinus in 59 (98.3%) patients. The maxillary sinus was the most commonly involved sinus in49 (81.7%) patients, followed by anterior and posterior ethmoidsin 41 (68.3%), frontal in 24 (40%) patients and sphenoid in 12 (20%) patients. The mean Lund-Mackay CT sinus score as assessed independently by two of the authors were 16.78 ±SD 3.76 (range from 9 to 24). The median score was 16 and mode was 14 (Table 2).

**Table 2:** The association between overall disease severity score and Lund – Mackay grading of paranasal sinus findings on CT scan

Visual Analogue Score	≤4 Low scores	5 – 7 intermediate scores	≥8 high scores
Lund- Mackay grading			
0 (No opacity)	1	0	0
1 (partial opacity)	11	20	4
2 (Total opacity)	4	4	16

Patients with mild/low disease severity scores had CT scores ranging from 10 to 22, patients with moderate/intermediate disease severity scores had CT scores ranging from 12 to 24 whereas patients with high/severe disease severity score had CT scores ranging from 9 to 24. Statistical analysis showed no significant correlation between the overall disease severity scores and the Lund-Mackay CT scores (r = 0.195; p = 0.6). There was a significant correlation between CT scores and nasal discharge (r = -0.132; p = 0.03) but a weak correlation between CT scores and nasal obstruction (r = 0.193; p = 0.049) (Table 3). The remaining symptoms did not show any significant association with the CT score.

**Table 3:** The association between overall disease severity score and Lund – Mackay grading of osteo-meatal complex on CT scan

Visual Analogue Score	≤4 Low scores	5 – 7 Intermediate scores	≥8 High scores
Lund-Mackay grading
0 ( Patent)	12	5	1
2 ( Occluded)	4	19	19

Anatomic variations were found on the CT scans of 15 patients and these included 4 patients with Concha bullosa, one with paradoxical middle turbinate, two with haller cells, one with Onodi cells, one with ager nasi and six patients with deviated septum. There was no association between disease severity score and anatomical variations (p > 0.05).

## Discussion

Rhinosinusitis is a significant health problem with its rising prevalence corresponding with the increasing frequency of allergic rhinosinusitis worldwide. There were more males with the disease in this study. This agrees with the findings from other similar studies on chronic rhinosinusitis where the disease has been reported to have a higher male sex predilection [16, 17].

The symptomatology of patients in this study is similar to what had also been reported in the literature [1–3, 18, 19]. The clinical diagnosis of the patients in this study was based on the presentation of nasal discharge and nasal obstruction in all the patients which were two major diagnostic criteria [2]. The maxillary sinus was identified as the most involved paranasal sinus by the disease. This finding is similar to what had been reported in other previous studies [20, 21]. The role of maxillary sinus in chronic rhinosinusitis is very important and the sinus should be considered while managing the disease. Ethmoidal sinus was the second most involved paranasal sinus in this study and mucosal thickening in the sinus is highly associated with smell alteration.

In this study, the authors have used a simple staging system to grade symptom severity of chronic rhinosinusitis. The patients had different perspective of their symptoms, the severity and effect on their daily routine and sleep. The mean symptom severity scores were higher for nasal obstruction and nasal discharges than for other symptoms. These were major criteria already recommended by the Rhinosinusitis Task Force of the American Academy of Otolaryngology, Head and Neck Surgery (AAO-HNS) for the diagnosis of chronic rhinosinusitis [2]. The effects of chronic rhinosinusitis on the quality of life of the patients were well documented in the literature. In this present study, about 1/3rd of the patients reported that the disease severely affected their daily routine and sleep while less than 1/3rd reported mild affectation. Disturbance of sleep will affect performance at school and productivity at work with significant financial and economic loss. The effect of CRS on the quality of life of patients has been reported in the literature [22]. Therefore, CRS should be considered a public health problem and given appropriate medical attention in a nation's health policy.

Normal nasal physiology is dependent on the healthy condition of sinus ostiafor proper drainage and ventilation of the paranasal sinuses [23, 24]. These ostia are located in an area on the lateral wall of the nose calledostiomeatal complex. Ostiomeatal complexreceives the drainage of most paranasal sinuses and when occluded, it is an important factor in the pathogenesis of chronic rhinosinusitis [2, 3]. Previous similar studies have documented other local, systemic and environmental factors as more important factors in the pathogenesis of chronic rhinosinusitis [25–27]. The finding of patency of ostiomeatal complex in 30% of patients in this study supports the contributions of other factors,in addition to occlusion of ostiomeatal complex, in the etiopathogenesis of chronic rhinosinusitis. Specifically, anatomical variations which redirect nasal airflow or narrow the ostiomeatal complex have been considered important factors in the pathogenesis of chronic rhinosinusitis [28]. The anatomic variations identified in this study are similar to what other studies have reported [25–28]. Differentstudies have reported on the association ofanatomic variations withthe pathogenesis of chronic rhinosinusitis, while few studies documented no specific association [21, 25–27], a study reported a causal association of concha bullosa with chronic rhinosinusistis [29]. This present study also found no association between anatomic variations and severity of the disease (p > 0.05). It was also observed that there was no difference in the presence of concha bullosa and septal deviation between patients with low and high disease severity scores.

CT scan has been used by clinicians to make diagnose, delineate disease extentand identify the involved paranasal sinus as well as any anatomical abnormalities [30]. In addition, it serves as a road map for the surgeons during endoscopic sinus and base of skull surgeries [31]. This present study investigated its role as a proxy for symptom or overall disease severity and vice versa. It found an association between CT scan score and nasal discharge and obstruction. This agrees with the findings of statistically significant correlation between symptoms and CT score from other similar studies [22, 32]. Contrary to the above findings, few studies exist which demonstrated no correlation between CT score of Lund Mackay and chronic rhinosinusitis [33, 34]. Because of failure of previous studies to arrive at a uniform conclusion, the authors suggest the consideration of disease severity score in the assessment of chronic rhinosinusitis in addition to clinical symptoms and CT scan. Although there appears to be no association between the CT score and overall disease severity score, their combination can provide an ideal way to help clinicians understand and better grade the patients’ disease and thus prioritize them for therapy. The lack of correlation for other symptoms of chronic rhinosinusitis with CT score of Lund Mackay in this present study may be due to their absence in a large number of patients with positive CT scan findings of chronic rhinosinusitis as well as symptomatic patients with minimal sinus disease on CT scan.

This present study found mucosal thickening in one or more paranasal sinuses of 98% patients with chronic rhinosinusitis. This makes CT scan an important tool in the assessment of chronic rhinosinusitis. However, this finding contradicts the report that CT scan is inadequate as a sole instrument for the assessment and grading of chronic rhinosinusitis from a similar study [33]. Few studies have also documented incidental findings of mucosal thickening and isolated polyps on the CT scan of patients without symptoms of chronic rhinosinusitis [35, 36]. Therefore, for better assessment of morbidity and activity of chronic rhinosinusitis, disease severity should be included.

## Conclusion

This study showed that CT scan scores can help clinicians to predict severity of symptom for nasal obstruction and discharge but not for other symptoms of chronic rhinosinusitis. However, there was no association of CT score with the overall disease severity score. This present study also found no association between anatomic variations and severity of the disease (p > 0.05).
